# A longitudinal study of gender differences in quality of life among Japanese patients with lower rectal cancer treated with sphincter-saving surgery: a 1-year follow-up

**DOI:** 10.1186/s12957-015-0485-x

**Published:** 2015-03-04

**Authors:** Yumiko Kinoshita, Akiko Chishaki, Rieko Kawamoto, Tatsuya Manabe, Takashi Ueki, Keiji Hirata, Mami Miyazono, Maki Kanaoka, Akiko Tomioka, Masahiro Nakano, Tomoko Ohkusa, Hisako Nakao, Masao Tanaka, Ryuichi Mibu

**Affiliations:** Department of Health Sciences, Graduate School of Medical Sciences, Kyushu University, 3-1-1 Maidashi, Higashi-ku, Fukuoka 812-8582 Japan; Japanese Nursing Association, 5-8-2 Jingu-mae, Shibuya-ku, Tokyo 150-0001 Japan; Department of Surgery and Oncology, Graduate School of Medical Sciences, Kyushu University, 3-1-1 Maidashi, Higashi-ku, Fukuoka 812-8582 Japan; International University of Health and Welfare, 1-7-4 Momochihama, Sawara-ku, Fukuoka 814-0001 Japan; School of Nursing, Fukuoka Prefectural University, 4395 Ita, Tagawa-city, Fukuoka 825-8585 Japan; Department of Nursing, Junshin Gakuen University, 1-1-1 Chikushigaoka, Minami-ku, Fukuoka 815-8510 Japan; Kirameki Project Carrier Support Center, Kyushu University Hospital, 3-1-1 Maidashi, Higashi-ku, Fukuoka 812-8582 Japan

**Keywords:** Gender differences, Gender-related factors, Prospective study, Quality of life, Rectal neoplasms, Sphincter-saving surgery, Symptoms

## Abstract

**Background:**

Up to 80% of patients with rectal cancer undergo sphincter-saving surgery, and almost 90% of them experience subsequent physical changes. The number of studies on gender differences in response to this surgery has increased, and the connection between gender and symptoms and patient outcomes has generated increasing interest. Nevertheless, little is known about the gender differences in quality of life and cancer-related symptoms. We examined gender differences and quality of life changes over a 1-year period among patients with lower rectal cancer who were treated with sphincter-saving surgery.

**Methods:**

Patients (men = 42; women = 33) completed a self-administered questionnaire on their quality of life and related factors before surgery and 1, 6, and 12 months afterwards. The questionnaire was developed by the European Organization for Research and Treatment of Cancer (EORTC QLQ-C30/CR-38).

**Results:**

Scores on physical, role, and social functioning and global health status/quality of life decreased 1 month after surgery, improved after 6 months, and returned to baseline within 12 months, with the exception of social functioning in men. Factors related to quality of life changed after surgery and differed between men and women. Women’s global health status/quality of life was affected by fatigue, weight loss, defecation problems, and future perspective, while that of men was affected by fatigue, weight loss, future perspective, and role functioning, which was affected by pain, defecation problems, and financial difficulties.

**Conclusions:**

Gender differences should be considered when predicting the quality of life of cancer patients undergoing surgery. Identifying gender differences will help health care providers anticipate the unique needs of patients undergoing surgery for rectal cancer.

## Background

Rectal cancer is one of the most common malignant tumors, and sphincter-saving surgery (SSS) and abdominoperineal resection (APR) have been widely used to treat rectal cancer. The psychological needs of patients undergoing APR are of greater concern than those undergoing SSS because people can imagine the handicap caused by this major change, including the care of permanent stoma after APR. Due to the decreased quality of life (QOL) that accompanies APR with a permanent stoma, it tends to be avoided, and low anterior resection (LAR) is chosen instead [1].

Disorders of sexual, urinary, and bowel function might result from surgery for rectal cancer, and the dysfunction may influence the patient’s QOL. Traa *et al.* reported that treatment groups of 439 patients with rectal cancer had lower QOL and sexual functioning compared to the general population [2]. Chambers *et al.* identified a permanent stoma as a risk factor for a lower QOL [3]. That study reported that rectal cancer patients having SSS might experience symptoms affecting their QOL that are different from those of stoma patients. However, the study’s conclusions did not appear in a meta-analysis conducted by Cornish *et al.* or in a systematic review by Pachler and Wille-Jørgensen published in the Cochrane Database [4,5].

In recent years, the demand for SSS has increased in patients with middle or low rectal cancer because of the esthetic appeal of the lower-sited anastomosis due to the development of surgical techniques. Although patients undergoing SSS have defecation problems, they request surgery to avoid having a permanent stoma. The techniques of SSSs include LAR, ultra-low anterior resection (ULAR), and intersphincteric resection (ISR). LAR is commonly used when the cancer has affected the middle rectum. The ULAR includes anastomoses at 2 cm from the dentate line. The ISR is the ultimate SSS for very low rectal cancer, which includes a partial resection of the internal anal sphincter [6].

Bryant *et al.* reported that up to 80% of rectal cancer patients undergo SSS [7]. Up to 90% of patients who undergo SSS will experience changes in their bowel habits, changes that range from heightened bowel frequency to fecal incontinence or evacuatory dysfunction. Moreover, sexual dysfunction and dysuria frequently occur after surgery for lower rectal cancer.

Desnoo and Faithfull, Nikolette *et al.*, and Landers *et al.* described the experience and management of defecation problems in patients after SSS in three qualitative studies and argued for the need to strengthen support for them [8-10]. A grasp of the problems associated with anorectal symptoms and QOL after SSS is important for the care of patients with lower rectal cancer. There are previous studies on APR (AR and LAR); however, estimates of QOL after APR and SSS have been controversial [5]. Few studies have focused on the QOL of patients undergoing SSS. An analysis of data on SSS and QOL using the European Organization for the Research and Treatment of Cancer Core Quality of Life Questionnaire (EORTC QLQ-C30 and CR38) found a higher QOL in patients treated with the high anterior resection than those treated with LAR and APR [1]. Therefore, LAR patients require more attention because of their lower QOL scores compared with the APR patients. Although patients with a low anastomosed line 3 cm apart from the anal verge had incontinence of gas and feces, no difference in QOL was found on the EORTC QLQ-C30/CR38, compared with patients with higher anastomosis [11].

Camilleri-Brennan and Steele observed that patients’ QOL after rectal cancer surgery deteriorated immediately and changed over time [12]. Schmidt *et al.* found that the majority of QOL scores fell below baseline in the early postoperative period [13]. In a prospective study, Andersson *et al.* compared the QOL of a patient subset from a randomized trial (*N* = 385) 12 months after laparoscopic versus open surgery for rectal cancer [14]. Changes in EORTC QLQ-C30 and CR38 were not significantly different between the groups. Physical, role, and social functioning and fatigue, as measured by the EORTC QLQ-C30, revealed a substantial deterioration 1 month after surgery. All of the functional and symptom scores improved after 6 months and returned to baseline levels within 12 months. The early postoperative period is important for patients to accept changes caused by surgery, and thereafter, to accommodate to postoperative life. However, gender differences were not considered.

Little is known about the gender differences in QOL, and studies on cancer-related symptoms have yielded conflicting results [15]. In a study by Hjermstad *et al.*, Norwegian women reported lower functional status and global health status/QOL than did their male counterparts, and Schwarz and Hinz reported similar findings in the general German population [16,17]. Fayers *et al.* concluded that the effects of age and gender based on reference data from the EORTC QLQ-C30 (+3) must be considered when interpreting data on the QOL of cancer patients [18]. Little is known about the association between patients’ gender and their differing perceptions of their QOL after surgery for rectal cancer. Schmidt *et al.* reported that QOL scores often decreased significantly during the period soon after surgery [19]. From the third month after surgery, global health status/QOL, emotional functioning, and physical functioning improved. Women had significantly inferior scores on global health status/QOL and physical functioning and higher scores on treatment strain and fatigue. Men declared difficulties with sexual enjoyment; gradually, sexual problems caused high levels of strain in men, with scores inferior to baseline levels in the period soon after surgery.

To our knowledge, there are no existing studies investigating gender differences in the longitudinal QOL changes among patients with lower rectal cancer after only SSS. Therefore, the purpose of this study was to examine gender differences in the changes in QOL before and after SSS in patients with lower rectal cancer.

## Methods

### Study population and data collection

The inclusion criteria for participation in this study were patients who (1) had the psychological capability of completing the QOL questionnaires, (2) were adults (over 20 years of age), (3) had clinical stage I to IIIb cancer but not metastasis, and (4) had no postoperative complications. Patients with lower rectal cancer underwent primary surgery at the study hospitals between November 2008 and March 2013.

A researcher explained the study protocol to the patients. The patients who agreed to participate in the study completed the self-administered questionnaires. The self-administered questionnaires were mailed or hand delivered to the participants immediately before the surgery in the hospital and at 1-, 6-, and 12-month intervals following their operations. Clinical data were gathered from each institutional database. All collected questionnaires were coded and stored in a secure location to protect the patients’ privacy. Eighty-eight patients who had SSS provided a written consent; 85 agreed to participate in the study. Postoperative complications showed anastomotic leakage in two cases: men and women with ISR and the ULAR group. Accordingly, we excluded all participants in these cases, and 75 completed the entire set (four administrations) of questionnaires. Ethical approval was obtained from the review boards of the university and hospital in which the study was conducted.

### Therapy

The operations were performed by laparoscopic surgery. Intestinal continuity was restored by performing a straight anastomosis. Diversion was accomplished with a temporary loop ileostomy. The diversion was used selectively in patients, based on whether the surgeon thought they would be at high risk for an anastomotic leak. Twenty-four (18 men) of 75 patients had temporary loop ileostomies after ISR and ULAR. The median length of time for the closure of the loop ileostomies, from surgery to stoma reversal, was 167.5 days: range = 17 to 425 days (men: 176.0 days, range = 17 to 425 days; women: 166.5 days, range = 108 to 292 days).

Neoadjuvant radio-chemotherapy is the standard therapy for patients with locally advanced rectal cancer in Western countries. However, in Japan, surgery without neoadjuvant radiotherapy is the standard procedure for patients with stage T3 to T4 lower rectal cancer because lateral lymph node dissection is a standard procedure as opposed to radiotherapy in Japan. Only seven (6 men) patients from ISR and ULAR group received neoadjuvant and adjuvant chemotherapy, with the strategy of neoadjuvant chemotherapy being the facilitation of sphincter preservation by potential downstaging. Moreover, in general, patients begin a 6-month course of adjuvant chemotherapy within 2 months after surgery. Some patients might refuse chemotherapy because of adverse events.

### Questionnaires

The European Organization for Research and Treatment of Cancer (EORTC) Quality of Life Questionnaire (QLQ)-C30 version 3.0, also referred to as the EORTC QLQ-C30, is a questionnaire measuring the QOL of patients who have cancer [18,20,21]. It includes a global health status/QOL scale, five multi-item functional scales, and nine multi-item symptom scales. The five multi-item functional scales measured physical, role, emotional, cognitive, and social functioning, and the nine multi-item symptom scales measured fatigue, nausea and vomiting, pain, dyspnea, insomnia, appetite loss, constipation, diarrhea, and financial difficulties.

The Japanese version of the EORTC QLQ-CR38 assesses QOL in colorectal cancer patients and includes four functional scales (body image, sexual functioning, sexual enjoyment, and future perspective) and eight symptom scales/items (micturition problems, chemotherapy side effects, gastrointestinal tract symptoms, male sexual problems, female sexual problems, defecation problems, stoma-related problems, and weight loss) [18,20,22]. We obtained permission for the use of this questionnaire from EORTC Quality of Life Group (Brussel, Belgium). Scores range from 0 to 100. Higher scores on global health status/QOL and the five multi-item functional scales indicate higher levels of functioning, and higher scores on the symptoms scales represent higher levels of symptoms. Osoba *et al.* suggested that a change of five to ten points in a patient’s score indicates a minimal change, whereas a change of more than 20 points indicates a substantial change [23].

Cronbach’s α was calculated for the following measures: global health status/QOL (0.738 to 0.916), physical functioning (0.849 to 0.881), role functioning (0.925 to 0.952), emotional functioning (0.738 to 0.916), cognitive functioning (0.624 to 0.800), and social functioning (0.802 to 0.850).

### Statistical methods

Statistical analyses of the scores on the EORTC questionnaires were performed in accordance with the scoring manual and involved the transformation of raw scores to a linear scale ranging from 0 to 100. Mann-Whitney U and chi-square tests were used to make comparisons between the groups [18]. Data from the follow-up study were analyzed using a repeated-measure ANOVA (gender × time factor) and the Bonferroni test. This study aimed to include at least 46 participants/arm, which was expected to yield power ≥0.80, based on *α* ≤ 0.05, and assuming a medium effect size (i.e., *f* = 0.25; Cohen, 1977).

Spearman’s correlation coefficient was used to examine the relationship between global health status/QOL and the functional and symptom scales (EORTC QLQ-C30). Spearman correlations were performed to analyze any relationships between the predictors and QOL. Multiple linear regression analysis (stepwise) was performed on the independent predictors of EORTC QLQ-C30/CR38 scores. Variables correlated with global health status/QOL (*P* < 0.1) were included in the Spearman correlations as independent variables predictive of QOL magnitude in women and men separately. Separate multiple linear regression analyses (stepwise) were conducted for men and women to identify the independent variables (EORTC QLQ-C30 and CR38) predictive of global health status/QOL (dependent variable). The level of statistical significance was set to 0.05. SPSS (Version 21.0 for Windows, Tokyo, Japan) and was used for all statistical analyses.

## Results

The mean ages of the final sample of 42 men and 33 women were 60.6 and 57.5 years, respectively (Table 1). There was no significant difference in age according to gender (*P* = 0.160), and no significant differences in clinical information (clinical stage, surgery type, chemotherapy, and radiation) or social information (occupational status, marital status, and living with others) between the two groups. However, temporary ileostomy rates was found to be statistically significant across the gender (men: 42.9% versus women: 18.2%, *P* = 0.023). Overall, treatment duration was also statistically significant (men: 437.9 ± 126.5 days (range = 365 to 790 days) versus women 391.6 ± 65.8 days (range = 365 to 657 days), *P* = 0.037).

**Table 1:** **Demographic and rectal cancer-related information (** ***N*** **= 75)**

	**Men (** ***n*** **= 42)**		**Women (** ***n*** **= 33)**		***P*** **value**
*Mean age* (*SD*) *in years*	60.6	(10.6)	57.5	(7.6)	0.160
*Occupational status* (*%*)					
Employed full or part-time	14	33.3	15	45.5	0.285
Unemployed	28	66.7	18	54.5	
*Marital status*					
Married	41	97.6	32	97.0	1.000
Other	1	2.4	1	3.0	
*Living with others*					
No	1	2.4	4	12.1	0.093
Yes	41	97.6	29	87.9	
*Clinical stage* (*tumor node metastasis*)					
I	22	52.4	18	54.5	0.488
II	6	14.3	7	21.2	
IIIa	9	21.4	3	9.1	
IIIb	5	11.9	5	15.2	
*Surgery type*					
ISR (temporary ileostomies)	11 (11)	26.2	5 (4)	15.2	0.099
ULAR (temporary ileostomies)	16 (7)	38.1	8 (2)	24.2	
LAR	15	35.7	20	60.6	
*Neoadjuvant chemotherapy*					
No	36	88.1	32	97.0	0.096
Yes	6	11.9	1	3.0	
*Adjuvant chemotherapy*					
No	24	57.1	21	63.6	0.371
Yes	18	42.9	12	36.4	
*Radiation*					
No	42	100.0	33	100.0	1.000
Yes	0	0.0	0	0.0	

Surgery: *ISR* intersphincteric resection, *ULAR* ultra-low anterior resection, *LAR* low anterior resection.

### Changes in EORTC QLQ-C30 and CR38 scores

Changes in participants’ scores on the EORTC QLQ-C30 and the EORTC QLQ-CR38 are presented in Tables 2 and 3, respectively. Before surgery, the patients’ scores on global health status/QOL and social functioning were more than ten points lower than the reference data (general German population). We found a small decrease in global health status/QOL 1 month after surgery and an improvement after 6 months. Physical, role, and social functioning deteriorated significantly 1 month after surgery, improved after 6 months, and returned to baseline levels by 12 months, with the exception of role and social functioning in men. Role functioning showed the largest decrease 1 month after surgery, and scores were more than 20 points below baseline for both genders. However, there were no significant changes in emotional or cognitive functioning in either group during the 12 months following surgery.

**Table 2:** **EORTC QLQ-C30v3 scores by time and gender (** ***N*** **= 75)**

**Questionnaire time points**																
	**Reference data General German population**	**Men (** ***n*** **= 42)**				**Reference data General German population**	**Women (** ***n*** **= 33)**									
		**Before**	**Postoperative time (month)**				**Before**	**Postoperative time (month)**			**Main effect of time**	**η** ^**2**^_**p**_	**Main effect of gender**	**η** ^**2**^_**p**_	**Time-by-gender interaction**	**η** ^**2**^_**p**_
		**(T0)**	**1 (T1)**	**6 (T2)**	**12 (T3)**		**(T0)**	**1 (T1)**	**6 (T2)**	**12 (T3)**	**(T0, T1, T2, T3)**					
	**Men**					**Women**										
	**Mean**	**Mean**	**Mean**	**Mean**	**Mean**	**Mean**	**Mean**	**Mean**	**Mean**	**Mean**	***P*** **values**		***P*** **values**		***P*** **values**	
	**SD**	**SD**	**SD**	**SD**	**SD**	**SD**	**SD**	**SD**	**SD**	**SD**						
*EORTC QLQ-C30v3*																
*Global health status/QOL*	72.7	61.5	53.8	64.5	64.7	69.2	57.8	56.1	68.2	70.7	<0.001	0.099	0.566	0.005	0.411	0.013
	22.2	21.1	25.2	24.2	20.2	21.9	23.5	25.5	20.0	21.8	T1 < T2** T1 < T3***					
*Functional scales*																
Physical functioning	92.0	92.2	82.9	90.6	90.3	88.7	87.9	81.6	89.1	90.5	<0.001	0.122	0.447	0.008	0.584	0.008
	15.6	14.4	16.8	11.0	12.0	15.9	26.2	14.4	9.8	10.9	T0 > T1**					
											T1 < T2** T1 < T3***					
Role functioning	89.8	84.5	63.5	75.4	76.6	86.6	77.8	57.6	79.8	84.3	<0.001	0.209	0.997	0.000	0.060	0.036
	21.7	20.6	30.4	27.4	23.3	23.7	27.2	30.1	19.0	19.1	T0 > T1***					
											T1 < T2*** T1 < T3***					
Emotional functioning	81.8	78.3	74.8	79.9	80.5	76.3	76.5	79.6	85.4	80.3	0.170	0.024	0.559	0.005	0.456	0.011
	18.8	20.6	25.3	19.9	18.8	22.2	18.3	24.6	14.1	23.7						
Cognitive functioning	92.7	83.7	78.0	79.4	78.2	90.1	81.3	83.8	82.8	85.4	0.845	0.003	0.339	0.013	0.130	0.026
	15.0	17.8	23.0	18.3	20.3	18.4	19.9	18.4	18.8	17.1						
Social functioning	92.0	77.8	65.3	73.4	73.4	90.3	77.3	68.7	82.3	85.9	<0.001	0.094	0.187	0.024	0.141	0.025
	18.3	27.2	29.9	24.7	23.9	20.1	25.3	25.2	20.4	20.0	T0 > T1*		At 12 months after surgery: men < women *P* = 0.030			
											T1 < T2** T1 < T3**					
*Symptom scales/items* (*#*)																
Fatigue	14.0	23.3	35.6	22.8	23.8	19.5	28.3	35.7	29.0	25.9	<0.001	0.090	0.290	0.015	0.632	0.007
	0.3	18.3	23.5	16.3	17.6	23.1	21.7	20.7	16.9	20.5	T0 < T1*					
											T1 > T2** T1 > T3*					
Nausea and vomiting	1.8	0.8	3.6	1.2	1.6	3.6	3.0	6.6	3.5	3.0	0.063	0.038	0.163	0.026	0.860	0.002
	7.6	3.6	10.8	4.3	6.2	11.4	8.8	18.1	10.0	7.7						
Pain	13.0	14.7	26.2	16.3	17.1	17.2	20.2	18.7	16.2	11.6	0.064	0.035	0.649	0.003	0.148	0.025
	23.1	21.5	29.9	24.0	25.4	25.3	23.5	20.7	23.4	18.4						
Dyspnea	6.9	12.2	8.1	8.1	7.3	9.1	9.1	7.1	6.1	10.1	0.455	0.012	0.784	0.001	0.616	0.080
	18.5	24.5	16.3	20.8	15.8	21.6	17.2	20.0	13.1	15.6						
Insomnia	13.0	23.0	31.8	22.2	26.2	19.1	27.3	31.3	28.3	25.3	0.301	0.016	0.681	0.002	0.741	0.005
	24.4	29.9	31.2	32.7	35.0	29.0	29.4	35.3	30.2	23.6						
Appetite loss	4.2	6.4	10.3	7.2	3.2	6.3	17.2	29.3	16.2	12.1	0.005	0.064	0.002	0.130	0.346	0.015
	14.0	13.3	26.6	18.8	9.9	17.4	27.8	35.1	27.8	20.1	T1 > T3**		Men < Women			
Constipation	2.5	28.6	28.4	23.0	23.0	4.3	35.4	26.3	33.3	28.3	0.467	0.011	0.296	0.015	0.485	0.011
	11.8	32.6	30.9	29.9	32.5	14.9	36.3	27.3	25.0	22.3						
Diarrhea	2.5	23.0	23.4	22.2	23.0	3.1	23.2	24.2	20.2	14.1	0.402	0.013	0.504	0.006	0.448	0.012
	10.4	25.0	26.3	21.7	22.7	12.6	22.8	25.4	26.3	18.7						
Financial difficulties	5.5	17.5	28.8	19.8	22.2	6.3	13.1	24.2	15.2	16.2	0.007	0.057	0.336	0.013	0.989	<0.001
	17.8	29.7	26.4	25.6	30.1	18.6	24.9	33.6	23.7	26.5	T0 < T1* T1 > T2**					

Higher scores on functional scales and overall QOL scale indicate higher levels of function. Higher scores on symptoms scales or single items (#) indicate higher levels of symptoms or problems.

*P < 0.05; **P < 0.01; ***P < 0.001.

**Table 3:** **EORTC QLQ-CR38 scores by time and gender**

**Questionnaire time points**									**Main effect of time**		**Main effect of gender**		**Time-by-gender interaction**	
	**Men (** ***n*** **= 42)**				**Women (** ***n*** **= 33)**									
	**Before**	**Postoperative time (month)**			**Before**	**Postoperative time (month)**				**η²**_**p**_		**η²**_**p**_		**η²**_**p**_
	**(T0)**	**1**	**6**	**12**	**(T0)**	**1**	**6**	**12**	**(T0, T1, T2, T3)**					
		**(T1)**	**(T2)**	**(T3)**		**(T1)**	**(T2)**	**(T3)**						
	**Mean**	**Mean**	**Mean**	**Mean**	**Mean**	**Mean**	**Mean**	**Mean**	***P*** **values**		***P*** **values**		***P*** **values**	
	**SD**	**SD**	**SD**	**SD**	**SD**	**SD**	**SD**	**SD**						
*Functional scales/items*														
Body image	85.2	71.6	80.4	78.8	82.2	75.8	76.4	81.8	0.006	0.064	0.992	0.001	0.298	0.017
	15.3	27.9	17.7	17.5	21.9	26.7	23.4	20.4	T0 > T1*					
									T1 < T2** T1 < T3**					
Future perspective	60.3	55.4	61.1	62.7	55.6	45.5	57.6	62.6	0.025	0.045	0.281	0.016	0.634	0.007
	25.8	34.8	26.5	26.8	28.5	33.2	19.2	21.7	T1 < T3*					
Sexual functioning	21.8	18.7	21.0	14.7	14.3	4.2	7.1	7.7	0.009	0.059	0.002	0.133	0.146	0.024
	21.6	19.6	17.7	14.8	19.1	9.8	15.3	16.7	T0 > T3*		Men > Women			
Sexual enjoyment	47.6	28.6	33.3	23.8	33.3	22.2	44.4	33.3	0.272	0.147	0.434	0.106	1.000	0.001
	26.2	29.9	27.2	25.2	22.5	19.3	19.3	22.5						
*Symptom scales/items* (*#*)														
Micturition problems	19.1	37.2	24.6	22.8	10.1	26.3	19.2	15.8	< 0.001	0.171	0.014	0.080	0.668	0.006
	21.7	27.5	17.1	17.2	12.5	21.1	17.6	15.5	T0 < T1*** T0 < T2*		Men > Women			
									T1 > T2* T1 > T3**					
Gastrointestinal tract symptoms	16.7	24.7	15.7	17.1	24.4	28.3	21.6	17.0	0.012	0.055	0.163	0.027	0.495	0.010
	19.3	20.1	12.9	15.7	27.6	30.0	14.8	2.5	T1 > T2* T1 > T3*					
Defecation problems	19.7	36.1	26.8	24.3	19.7	30.9	21.7	17.4	< 0.001	0.205	0.055	0.051	0.383	0.014
	15.3	18.1	11.4	15.6	14.0	14.5	11.2	11.0	T0 < T1***					
									T1 > T2*** T1 > T3***					
Weight loss	9.5	24.6	5.5	5.6	11.1	23.2	14.1	14.1	< 0.001	0.116	0.168	0.026	0.289	0.017
	18.5	28.6	12.6	12.6	16.0	28.2	26.4	23.6	T0 < T1**					
									T1 > T2*** T1 > T3***					

Higher scores on functional scales and overall QOL scale represent higher levels of functioning. Higher score on symptoms scales or single items (#) indicate higher levels of symptoms or problems.

*P < 0.05; **P < 0.01; ***P < 0.001.

A significant gender difference was found in social functioning 12 months after surgery, with men scoring significantly lower (73.4) than women (85.9) (see Table 2). Other QOL dimensions failed to differ significantly between the genders. Micturition problems were significantly worse for men after surgery. Measures of physical symptoms, such as appetite loss, indicated significant improvements (see Table 3). Sexual functioning was significantly reduced after surgery. Moreover, the erectile dysfunction rate increased after surgery, from 23.8% before to 54.8% 1 month after surgery, 42.9% 6 months after surgery, and 40.5% 12 months after surgery. Fatigue, financial difficulties, body image, micturition problems, defecation problems, and weight loss were worse 1 month after surgery. Baseline values for most of these items were restored by 1 year after the operation. Other factors did not change significantly during the 12 months after surgery.

### Correlations between global health status/QOL and EORTC QLQ-C30/CR38 factors

Before surgery, there was a significant positive correlation between global health status/QOL and all EORTC QLQ-C30 functional scales (see Table 4). One month after surgery, significant positive correlations were found between global health status/QOL and all EORTC QLQ-C30 functional scales, except cognitive functioning in women. In addition, when we divided the sample according to gender at 6 and 12 months after surgery, all of the significant relationships were found in the male group only. There were no significant correlations between global health status/QOL and EORTC QLQ-C30 functional scales among women, with the exceptions of physical functioning at 6 and 12 months after surgery and social functioning at 12 months after surgery.

**Table 4:** **Correlations between global health status/QOL scores and functional and symptom scales of EORTC QLQ-C30**

			**EORTC C30**													
			**Functional scales**					**Symptom scales/items (#)**								
			**Physical functioning**	**Role functioning**	**Emotional functioning**	**Cognitive functioning**	**Social functioning**	**Fatigue**	**Nausea and vomiting**	**Pain**	**Dyspnea**	**Insomnia**	**Appetite loss**	**Constipation**	**Diarrhea**	**Financial difficulties**
	Men	R	0.306	0.484	0.733	0.334	0.726	−0.604	−0.070	−0.479	−0.351	−0.561	−0.258	−0.270	−0.168	−0.528
Before Surgery		*p*	0.049	0.001	<0.001	0.031	<0.001	<0.001	0.658	0.001	0.023	<0.001	0.099	0.084	0.287	<0.001
	Women	R	0.543	0.680	0.779	0.516	0.646	−0.710	−0.017	−0.418	−0.283	−0.303	−0.365	−0.093	−0.393	−0.482
		*p*	0.001	<0.001	<0.001	0.002	<0.001	<0.001	0.927	0.015	0.111	0.087	0.037	0.607	0.024	0.005
1 month after surgery	Men	R	0.603	0.709	0.741	0.555	0.659	−0.642	−0.238	−0.656	−0.309	−0.524	−0.465	−0.082	−0.324	−0.336
		*p*	<0.001	<0.001	<0.001	<0.001	<0.001	<0.001	0.129	<0.001	0.046	<0.001	0.002	0.608	0.036	0.029
	Women	R	0.561	0.579	0.680	0.342	0.658	−0.784	−0.175	−0.354	−0.018	−0.669	−0.590	−0.099	−0.183	−0.436
		*p*	0.002	<0.001	<0.001	0.052	<0.001	<0.001	0.329	0.043	0.920	<0.001	<0.001	0.583	0.307	0.011
6 months after surgery	Men	R	0.650	0.672	0.479	0.548	0.525	−0.692	−0.082	−0.589	−0.252	−0.456	−0.249	−0.414	−0.109	−0.365
		*p*	<0.001	<0.001	<0.001	<0.001	<0.001	<0.001	0.608	<0.001	0.107	0.002	0.112	0.006	0.490	0.017
	Women	R	0.374	0.308	0.304	0.106	0.313	−0.611	−0.132	−0.168	−0.179	−0.290	−0.294	−0.176	−0.031	−0.314
		*p*	0.032	0.081	0.085	0.558	0.077	0.001	0.464	0.350	0.319	0.102	0.097	0.326	0.864	0.076
12 months after surgery	Men	R	0.650	0.672	0.479	0.548	0.525	−0.692	−0.082	−0.589	−0.252	−0.456	−0.249	−0.414	−0.109	−0.365
		*p*	<0.001	<0.001	0.001	<0.001	<0.001	<0.001	0.608	<0.001	0.107	0.002	0.112	0.006	0.490	0.017
	Women	R	0.469	0.328	0.338	0.335	0.532	−0.601	−0.405	−0.128	−0.053	−0.404	−0.470	−0.137	−0.091	−0.567
		*p*	0.006	0.063	0.054	0.057	0.001	<0.001	0.019	0.478	0.771	0.020	0.006	0.447	0.614	0.002

During the first year after SSS, a significant negative correlation was found between global health status/QOL and fatigue in both genders (see Table 5). Significant negative correlations were found between global health status/QOL and pain except 6 and 12 months after surgery in women. Significant negative correlations were found between global health status/QOL and financial difficulties except 6 months after surgery in women. Significant positive correlations were found between global health status/QOL and body image, except 6 months after surgery in women. Significant positive correlations were found between global health status/QOL and future perspective, except before and 6 months after surgery in women. Significant positive correlations were found between global health status/QOL and sexual functioning and sexual enjoyment within 1 and 6 months after surgery. Significant negative correlations were found between global health status/QOL and micturition problems within 6 and 12 months after surgery. Significant negative correlations were found between global health status/QOL and defecation problems except before surgery, 6 and 12 months after surgery in women.

**Table 5:** **Correlations between global health status/QOL scores and functional and symptom scales of EORTC QLQ-CR38**

			**EORTC QLQ-CR38**							
			**Functional scales**				**Symptom scales/items (#)**			
			**Body image**	**Future perspective**	**Sexual functioning**	**Sexual enjoyment**	**Micturition problems**	**Gastrointestinal tract symptoms**	**Defecation problems**	**Weight loss**
Before Surgery	Men	R	0.674	0.699	0.222	0.200	−0.137	−0.422	−0.349	−0.288
		*p*	<0.001	<0.001	0.158	0.397	0.387	0.005	0.025	0.064
	Women	R	0.585	0.167	0.144	0.546	−0.182	−0.377	−0.206	−0.355
		*p*	0.001	0.354	0.455	0.129	0.312	0.031	0.266	0.043
1 month after surgery	Men	R	0.565	0.685	0.240	0.575	−0.241	−0.528	−0.527	−0.568
		*p*	<0.001	<0.001	0.125	0.025	0.124	<0.001	<0.001	0.001
	Women	R	0.602	0.543	0.063	0.278	−0.166	−0.437	−0.430	−0.330
		*p*	<0.001	0.001	0.733	0.594	0.357	0.011	0.012	0.061
6 months after surgery	Men	R	0.450	0.501	0.193	0.281	−0.311	−0.393	−0.538	−0.159
		*p*	0.003	0.002	0.221	0.072	0.045	0.010	<0.001	0.315
	Women	R	0.205	0.247	0.549	0.415	−0.136	−0.083	−0.082	−0.100
		*p*	0.252	0.165	0.001	0.016	0.452	0.644	0.650	0.582
12 months after surgery	Men	R	0.450	0.501	0.193	0.281	−0.311	−0.393	−0.538	−0.159
		*p*	0.003	0.002	0.221	0.072	0.045	0.010	<0.001	0.315
	Women	R	0.352	0.469	0.211	0.218	−0.470	−0.213	−0.235	−0.154
		*p*	0.044	0.006	0.247	0.230	0.006	0.234	0.188	0.391

Males (*n* = 42), females (*n* = 33). Higher scores on functional scales and overall QOL scale indicate higher levels of functioning. Higher scores on symptoms scale or single items (#) indicate higher levels of symptoms or problems.

### Regression predicting global health status/QOL in men and women

**Figure 1:**
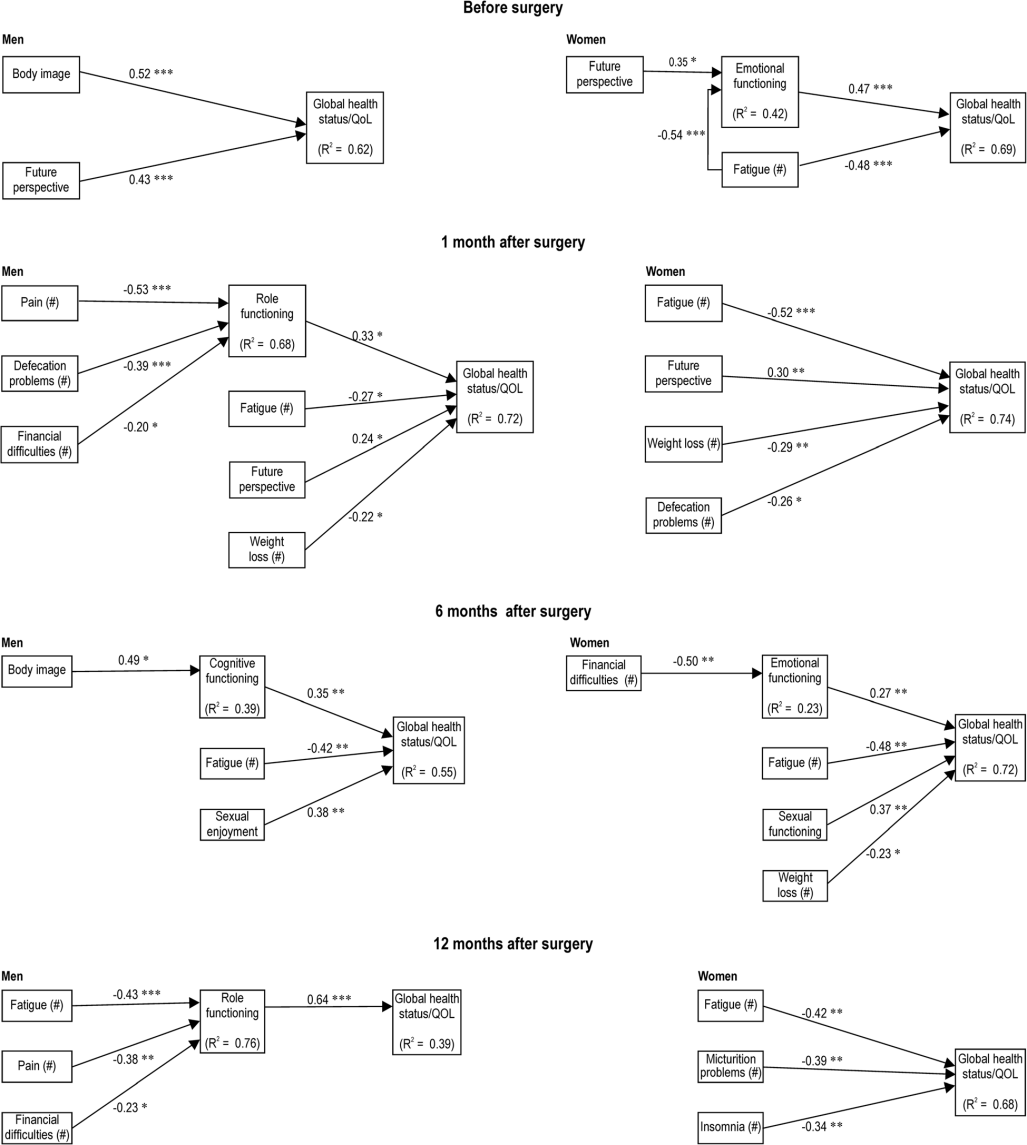
**Regression predicting global health status/QOL in men and women.** Higher scores on the functional scales and overall QOL scale indicate higher levels of function. Higher scores on the symptoms scales or single items (#) indicate higher levels of symptoms or problems. *P < 0.05; **P < 0.01; ***P < 0.001.

**Figure 2:**
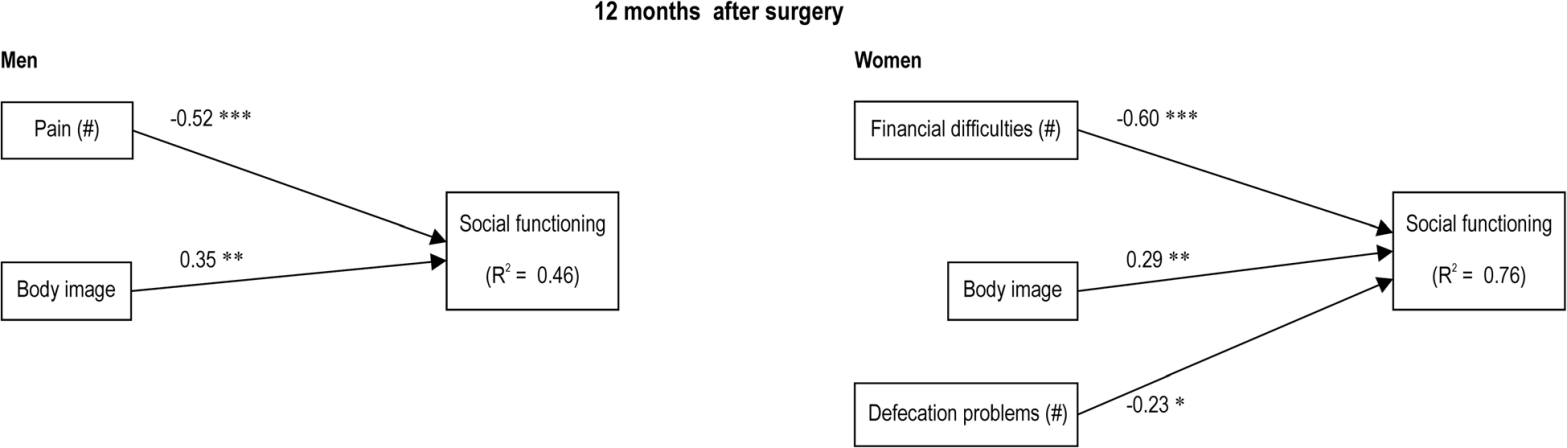
**Regression predicting social functioning at 12 months after surgery in men and women.** Higher scores on the functional scales and overall QOL scale indicate higher levels of function. Higher scores on the symptoms scales or single items (#) indicate higher levels of symptoms or problems. *P < 0.05; **P < 0.01; ***P < 0.001.

Separate multiple linear regression analyses (stepwise) were conducted for men and women to investigate predictors of global health status/QOL (Figure 1) and social functioning (Figure 2) 12 months after surgery. Before surgery, the global health status/QOL of men was significantly predicted by body image (*β* = 0.52) and future perspective (*β* = 0.43) (*F*(2, 39) = 52.62, *P* < 0.001, Adj. *R*^2^ = 0.62). Before surgery, the global health status/QOL of women was significantly predicted by emotional functioning (*β* = 0.47) and fatigue (*β* = −0.48) (*F*(2, 30) = 34.85, *P* < 0.001, Adj. *R*^2^ = 0.69), emotional functioning was significantly predicted by fatigue (*β* = −0.54), and future perspective (*β* = 0.35) (*F*(2, 30) = 12.39, *P* < 0.001, Adj. *R*^2^ = 0.42) before surgery.

One month after surgery, the global health status/QOL of men was significantly predicted by role functioning (*β* = 0.33), fatigue (*β* = −0.27), future perspective (*β* = 0.24), and weight loss (*β* = −0.22) (*F*(4, 37) = 26.55, *P* < 0.001, Adj. *R*^2^ = 0.72). Role functioning was significantly predicted by pain (*β* = −0.53), defecation problems (*β* = −0.39), and financial difficulties (*β* = −0.20) (*F*(3, 38) = 29.45, *P* < 0.001, Adj. *R*^2^ = 0.68). One month after surgery, the global health status/QOL of women was significantly predicted by fatigue (*β* = −0.52), future perspective (*β* = 0.30), weight loss (*β* = −0.29), and defecation problems (*β* = −0.26) (*F*(4, 28) = 23.34, *P* < 0.001, Adj. *R*^2^ = 0.74).

Six months after surgery, the global health status/QOL of men was significantly predicted by cognitive functioning (*β* = 0.35), fatigue (*β* = −0.42), and sexual enjoyment (*β* = 0.38) (*F*(3, 38) = 17.68, *P* < 0.001, Adj. *R*^2^ = 0.55). Cognitive functioning was significantly predicted by body image (*β* = 0.49) (*F*(1, 40) = 14.15, *P* < 0.001, Adj. *R*^2^ = 0.39). Six months after surgery, the global health status/QOL of women was significantly predicted by emotional functioning (*β* = 0.27), fatigue (*β* = −0.48), sexual functioning (*β* = 0.37), and weight loss (*β* = −0.23) (*F*(4, 28) = 21.21, *P* < 0.001, Adj. *R*^2^ = 0.72). Emotional functioning was significantly predicted by financial difficulties (*β* = −0.50) (*F*(1, 31) = 10.11, *P* < 0.001, Adj. *R*^2^ = 0.23).

Twelve months after surgery, the global health status/QOL of men was significantly predicted by role functioning (*β* = 0.64) (*F*(1, 40) = 27.53, *P* < 0.001, Adj. *R*^2^ = 0.39). Role functioning was significantly predicted by fatigue (*β* = −0.43), pain (*β* = −0.38), and financial difficulties (*β* = −0.23) (*F*(3, 38) = 44.50, *P* < 0.001, Adj. *R*^2^ = 0.76). Twelve months after surgery, the global health status/QOL of women was significantly predicted by fatigue (*β* = −0.42), micturition problems (*β* = −0.39), and insomnia (*β* = −0.34) (*F*(3, 29) = 22.58, *P* < 0.001, Adj. *R*^2^ = 0.68).

Twelve months after surgery, we found that men’s scores on social functioning were inferior to women’s scores. In men, social functioning was significantly predicted by pain (*β* = −0.52) and body image (*β* = 0.35) (*F*(2, 39) = 27.53, *P* < 0.001, Adj. *R*^2^ = 0.46). Twelve months after surgery, social functioning in women was significantly predicted by financial difficulties (*β* = −0.60), body image (*β* = 0.29), and defecation problems (*β* = −0.23) (*F*(3, 29) = 22.58, *P* < 0.001, Adj. *R*^2^ = 0.76).

## Discussion

This study attempted to show the longitudinal changes in the QOL of patients with lower rectal cancer 12 months after SSS and to reveal gender differences in QOL-related factors. Little is known about how patients’ QOL changes over time after SSS for lower rectal cancer or whether gender is associated with different perceptions of QOL.

Hjermstad *et al.* found that Norwegian women reported lower functional status and global health status/QOL than men did, which corresponded with findings in the general German population [16,17]. In our study, scores for both genders were similar before surgery, but most of the QOL scores changed after surgery, with men having lower scores than women. However, this may have been biased by longer overall duration treatment in men. Before surgery, global health status/QOL and social functioning scores exceeded the scores indicating ‘poor’ QOL of the reference (German) population by ten points. Global health status/QOL decreased 1 month after surgery and improved 6 months after surgery.

Physical, role, and social functioning deteriorated significantly 1 month after surgery, improved after 6 months, and returned to baseline levels within 12 months, with the exception of role and social functioning in men. Role functioning scores decreased conspicuously 1 month after surgery, more than 20 points below the pre-surgery scores for both genders. Women recovered 6 months after surgery, but men did not recover by 12 months after surgery. There were no significant changes in emotional or cognitive functioning during the 12 months following surgery for both groups. Andersson *et al.* reported that physical, role, and social functioning deteriorated 1 month after surgery, improved after 6 months, and returned to baseline levels within 12 months [14]. For both genders, role functioning scores on the EORTC QLQ-C30 decreased by more than 30 points 1 month after surgery from the baseline scores before surgery. This result was similar to our findings.

In our study, role, cognitive, and social functioning in men were more than ten points lower than the reference (German) population of men 12 months after SSS. Furthermore, men’s scores on the social functioning scale 12 months after surgery were significantly lower than women’s scores, and the difference was over ten points. Vironen *et al.* demonstrated that patients with rectal cancer after surgery had poorer social functioning than did a population control group [24]. Rauch *et al.* indicated that stoma patients unexpectedly reported improved social functioning in comparison to non-stoma patients, but a gender effect was not reported [25].

Schmidt *et al.* reported that gender differences after rectal cancer surgery included APR and SSS but not ISR surgery [13]. Women had significantly lower global health status/QOL and physical functioning scores, and they scored worse in terms of fatigue. Men reported reduced sexual enjoyment, and sexual problems were greater among men over time, as indicated by lower scores than those in the early postoperative period.

Our findings confirm that the QOL in patients with lower rectal cancer changes with time post-surgery. We found no significant differences in global health status/QOL, physical functioning, and fatigue across the gender. Twelve months after surgery, men’s scores on the social functioning scale were significantly lower than women’s scores. Appetite loss was significantly better, and micturition problems were significantly worse in men post-surgery. Sexual functioning is reduced, and the erectile dysfunction rate is increased post-surgery. Fatigue, pain, appetite loss, future perspective, body image, micturition problems, defecation problems, and weight loss significantly improved within 12 months after surgery. Changes in the other factors were not significant within 12 months after surgery.

da Silva *et al.* reported that female patients showed significant deterioration in their overall sexual functioning at 6 months after surgery, with a partial recovery by 12 months, and improvement in body image by 12 months [26]. In our study, the finding on body image was similar, but sexual function did not change significantly within 12 months post-surgery.

This study investigated the predictive ability of gender with regard to QOL among patients with lower rectal cancer who had undergone SSS within the last 12 months. Before surgery, our sample’s global health status/QOL was more than ten points lower than the reference data (German sample). To improve their global health status/QOL before surgery, men could pay more attention to their body image and sense of future perspective, while women could focus on their emotional functioning, fatigue, and future perspective. In our study, global health status/QOL decreased slightly in men 1 month after surgery. During this time, men might need help addressing problems related to role functioning, future perspective, and weight loss. Role functioning was significantly predicted by pain, defecation problems, and financial difficulties and showed the largest decrease (more than 20 points below baseline) 1 month after surgery. One month after surgery, women need help with fatigue, weight loss, defecation problems, and future perspective.

In our study, global health status/QOL improved 6 months after surgery; however, men scored worse than the reference population at 6 and 12 months after surgery. Men need help with cognitive functioning, fatigue, and sexual enjoyment to improve their global health status/QOL 6 months following surgery. Cognitive functioning was significantly predicted by body image. Within 12 months after surgery, men need help with role functioning, which was significantly predicted by fatigue, pain, and financial difficulties in our study.

Women appear to be affected by fatigue at all times and weight loss, defecation problems, sexual functioning, and micturition problems after surgery. Men appear to be affected by body image and future perspective before and 1 month after surgery. They were affected by fatigue, weight loss, pain, and sexual enjoyment 6 and 12 months after surgery. Hendren *et al.* reported that sexual dysfunction after surgery is common in both genders [27]. However, it does not seem to have a negative impact on their global health status/QOL. However, in our study, sexual dysfunction was related to global health status/QOL and seemed to be different in the follow-up months, which was likely due to the difference in follow-up times between Hendren’s (29 to 160 months after surgery) and our study [27].

Men’s scores on social functioning were lower than were those of women at 12 months after surgery, and social functioning was significantly predicted by pain and body image. Pain was significantly correlated with fatigue, insomnia, body image, future perspective, defecation problems, and financial difficulties. Rauch *et al.* suggested the consideration of the effects of residual pain and constipation on long-term QOL when creating and implementing a treatment plan [25]. Thus, pain control and establishing bowel functioning are important to QOL. In addition, in our study, most patients had severe diarrhea and incontinence, and so they were using antidiarrheal agent. Thus, we could not properly evaluate this effect. Significant differences were not found in global health status/QOL between genders, and only social functioning in men at 12 months after surgery was lower than that of the women. However, this may have been biased by longer overall treatment duration in men. These factors may have prolonged the recovery of their social functioning. On the other hand, patients with SSS are prone to developing a condition called ‘low anterior resection syndrome,’ which includes disturbances in bowel habits, ranging from increased bowel frequency to fecal incontinence or evacuatory dysfunction. Konanz *et al.* describe that incontinence and painful defecation are common problems [28].

Patients with SSS in our study were especially prone to develop this syndrome. In our study, defecation, sexuality, and micturition problems were correlated; however, the correlations with fatigue were stronger than those with QOL. Fatigue contributed to the prediction of QOL in both groups during the 1-year period. Management strategies for fatigue include psycho-educational interventions, exercise programs, and pharmacological treatments [29]. Engel *et al.* reported an improvement of EORTC QLQ-C30 and CR38 scores of rectal cancer patients treated with SSS in a 4-year prospective study on QOL [30]. Thus, we think that a long-term prospective study is necessary to improve the QOL of patients suffering from the adverse effects of lower rectal cancer surgeries.

## Conclusions

The present results suggest that gender is an important consideration in explaining the QOL between individuals. The identification of patients (in this case, gender) who are likely to experience negative changes in their QOL will help health care providers tailor treatment plans for individual patients for best results. Nursing interventions that are time sensitive and individualized should improve the QOL of patients suffering from the adverse effects of SSS for lower rectal cancer.

### Relevance to clinical practice

Our results indicate that it is necessary to improve patients’ global health status/QOL, conduct an assessment within 12 month after surgery, and help them to enhance their social functioning.

Differences in QOL scores and related functions/symptoms between genders suggest the need for a different approach towards improving their QOL by controlling contributory factors. Treatment of fatigue is essential towards this end. A considerable amount of information about permanent stoma is available, but less information about fatigue, defecation problems, sexual problems, and micturition problems associated with SSS is accessible to the general public. Therefore, more information about the patients’ experiences with SSS is needed. The necessary components of a support system should include the following: 1) information on coping with fatigue, defecation, sexual problems, micturition problems, and financial difficulties; and (2) psychological support to improve patients’ body image and future perspective. Services must be delivered in a timely fashion, in multiple stages, and planned while patients are awaiting SSS.

The information presented here provides valuable knowledge on the predictors and factors associated with QOL. With this knowledge, patients at risk for reduced QOL can be identified and treated accordingly. Future studies can use these findings, which can then become a database for relevant guidelines.

## Abbreviations

QOL: quality of life
EORTC QLQ-C30: The European Organization for Research and Treatment of Cancer
SSS: sphincter-saving surgery
ISR: intersphincteric resection
ULAR: ultra-low anterior resection
LAR: low anterior resection
